# Liver‐ and Immune‐Enriched Molecular Signatures Associated With Mortality in Older Adults

**DOI:** 10.1111/acel.70621

**Published:** 2026-07-15

**Authors:** Yi‐Long Huang, Wei‐Ju Lee, Pei‐Lin Lee, Li‐Ning Peng, Fei‐Yuan Hsiao, Liang‐Kung Chen

**Affiliations:** ^1^ Center for Healthy Longevity and Aging Sciences National Yang Ming Chiao Tung University Taipei Taiwan; ^2^ Department of Geriatric Medicine, School of Medicine National Yang Ming Chiao Tung University Taipei Taiwan; ^3^ Department of Family Medicine Taipei Veterans General Hospital Yuanshan Branch Yilan Taiwan; ^4^ Center for Geriatrics and Gerontology Taipei Veterans General Hospital Taipei Taiwan; ^5^ Graduate Institute of Clinical Pharmacy, College of Medicine National Taiwan University Taipei Taiwan; ^6^ Taipei Municipal Gan‐Dau Hospital (Managed by Taipei Veterans General Hospital) Taipei Taiwan

**Keywords:** aging, biomarkers, cohort study, cox regression, metabolomics, mortality risk, proteomics

## Abstract

Quantifying biological aging requires biomarkers that capture multisystem physiological decline beyond chronological age. We aimed to compare the prognostic performance of plasma proteomics, metabolomics, and conventional clinical risk factors for all‐cause mortality, and to characterize molecular pathways associated with mortality risk and age‐related physiological decline. Untargeted plasma proteomics and metabolomics were profiled in 848 community‐dwelling adults from the I‐Lan Longitudinal Aging Study (ILAS), followed for a mean of 8.5 years, during which 92 deaths occurred. Cox proportional hazards models were applied to identify mortality‐associated molecular features and their enriched biological pathways. We identified 79 proteins associated with all‐cause mortality (FDR < 0.1), predominantly liver‐derived and immune‐related. Pathway analysis revealed coordinated dysregulation across coagulation cascades, complement activation, oxidative stress responses, glucose metabolism, and bile acid metabolism. Elastic net regression was subsequently used to construct omics‐based mortality risk scores, which were further evaluated in an independent validation cohort from the Longitudinal Aging Study of Taipei (LAST). A 20‐protein mortality score achieved strong discrimination (*C*‐index 0.81), outperforming metabolite‐based models (*C*‐index 0.77) and clinical risk factors (*C*‐index 0.73) in the discovery cohort. In the external validation cohort, both proteomic‐ and metabolomic‐derived scores showed directionally consistent associations with mortality risk. Together, these findings identify liver‐ and immune‐enriched molecular signatures associated with mortality risk in older adults and support the utility of plasma multi‐omics profiling as a scalable precision tool for biological age assessment. The identified proteomic and metabolomic pathways may help inform future interventions targeting systemic aging and age‐related functional decline.

## Graphical abstract

Untargeted plasma proteomics and metabolomics identified molecular signatures associated with all‐cause mortality in older adults. A liver‐enriched proteomic profile was associated with mortality risk in the ILAS cohort and showed consistent associations in an independent validation cohort, supporting circulating proteomic profiles for mortality risk stratification.

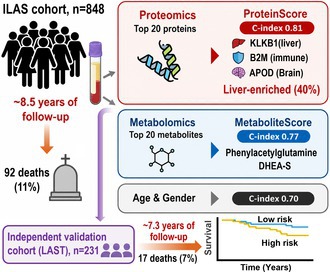

## 1 Introduction

Aging is a multifactorial process driven by interacting biological mechanisms, psychological states, social and environmental conditions that collectively determine an individual's phenotypic expression of aging (Lopez‐Otin et al. 2023). Although multiple methods have been developed to quantify aging, the field has increasingly entered an era in which biomarker‐based measures (e.g., epigenetic clocks, composite clinical biomarker scores, and metabolomic signatures) are used to operationalize “biological age” (Kroemer et al. 2025). While advances in healthcare and public health have substantially extended human lifespan, maximizing healthspan has emerged as a major priority (Masfiah et al. 2025). Accordingly, the integration of multi‐omics data to identify reliable biomarkers for the early detection of mortality and morbidity risk represents a promising approach in aging research (Shen et al. 2024). Recent advances in high‐throughput omics technologies offer unprecedented opportunities to identify prognostic biomarkers and elucidate pathophysiological pathways associated with aging. Although previous studies have identified individual metabolites and proteins associated with mortality, comprehensive multi‐omics investigations integrating proteomic and metabolomic data in well‐characterized aging cohorts remain limited. Furthermore, the relative contributions of circulating proteins versus metabolites in predicting mortality risk, as well as their biological origins and functional pathways, warrant systematic investigation.

The measurement of biological aging has transitioned from single biomarker approaches to a comprehensive multi‐omics paradigm that captures complexity of aging across multiple molecular layers. While epigenetic clocks—including Horvath's DNAm age (Horvath 2013), Hannum et al. (2013), PhenoAge (Levine et al. 2018), GrimAge (Lu et al. 2019), and DunedinPACE (Belsky et al. 2022)—have demonstrated robust chronological age prediction and mortality risk associations, they represented only one dimension of the aging process. Recent advances have integrated transcriptomic aging signatures that reflect age‐related gene expression changes (Fleischer et al. 2018; Peters et al. 2015), proteomic clocks such as those developed using plasma protein profiles that predict mortality and functional decline (Lehallier et al. 2019; Tanaka et al. 2018), and metabolomic biomarkers capturing systemic metabolic shifts during aging (Chaleckis et al. 2016; Robinson et al. 2020). The Biomarkers of Aging Consortium has emphasized the critical need for multimodal integration, proposing frameworks that combine epigenetic, proteomic, metabolomic, and physiological measurements to comprehensively assess biological age and validate longevity interventions (Moqri et al. 2023; Moqri, Herzog, et al. 2024). Emerging pan‐omics approaches are revealing interconnected regulatory networks underlying aging, including integrative analyses of DNA methylation and RNA expression in human blood (Moqri et al. 2026), enabling more precise identification of intervention targets and personalized risk stratification. This systems biology perspective acknowledged that aging was not quantified by any single molecular layer but rather emerged from the dynamic interplay across genomic, epigenomic, transcriptomic, proteomic, and metabolomic dimensions—a paradigm shift that promised to accelerate the translation of geroscience discoveries into clinical applications for extending healthspan and treating age‐related diseases (Kennedy et al. 2014).

In this study, we conducted untargeted proteo‐metabolomic profiling in healthy community‐dwelling middle‐aged and older adults with the mean follow‐up of 8.5 years to comprehensively identify plasma biomarkers associated with all‐cause mortality. We further characterized the biological pathways and tissue origins of mortality‐associated molecular signatures, developed and validated predictive models for mortality risk stratification, and compared the prognostic performance of protein‐based, metabolite‐based, and clinical variable‐based approaches across an independent cohort.

## 2 Results

### 2.1 Study Population

Of the 848 individuals with metabolomics and proteomic data in the I‐Lan Longitudinal Aging Study (ILAS) cohort, the mean age at enrollment was 63.8 ± 9.2 years and 53% were women. Over a mean follow‐up of 8.5 (SD 1.2) years, 92 participants (10.8%) died. Comparisons of clinical characteristics stratified by survival status and sex were presented in Table 1. Compared to survivors, deceased participants were older at baseline and more likely to be men (*p* = 0.0539). In both sexes, those who died had lower educational attainment, weaker grip strength, and higher disease burden (defined by Charlson Comorbidity Index) (Table 1). In addition, deceased participants were also more likely to be current smokers. Sex‐specific differences emerged for comorbidities: women who died had significantly higher prevalence of diabetes, hypertension, and congestive heart failure compared to female survivors, whereas these differences were not statistically significant in men.

**TABLE 1:** Baseline characteristics of participants from the I‐Lan Longitudinal Aging Study (ILAS) included in the metabolomics and proteomics study.

	Total survived	Total deceased	*p*	Female survived	Female deceased	*p*	Male survived	Male deceased	*p*
Participants, *n*	756	92		409	40		347	52	
Age, years	63.0 (8.7)	70.4 (10.4)	1.56E‐09	62.3 (8.4)	70.6 (10.3)	8.53E‐09	63.8 (9.0)	70.4 (10.6)	3.06E‐06
Female, %	54	43	0.0539	100	100	—	0	0	—
Education, years	6.7 (5.1)	4.6 (4.5)	1.95E‐04	5.9 (5.0)	3.7 (4.6)	8.22E‐03	7.7 (5.1)	5.3 (4.4)	1.71E‐03
BMI	24.9 (3.7)	25.1 (3.7)	0.515	24.6 (3.7)	25.7 (4.0)	0.081	25.2 (3.6)	24.7 (3.4)	0.368
Grip strength	28.7 (9.5)	25.5 (8.8)	1.94E‐03	22.5 (5.2)	19.4 (5.1)	2.65E‐04	36.0 (8.0)	30.2 (8.2)	1.65E‐06
Smoking status, %	15.2	23.9	0.0323	2.2	7.5	0.0473	30.5	36.5	0.3851
Alcohol status, %	31.7	31.5	0.9652	18.1	7.5	0.0898	47.8	50.0	0.7711
CCI	1.0 (1.2)	1.8 (1.4)	3.81E‐07	1.0 (1.2)	2.0 (1.2)	6.08E‐06	0.9 (1.1)	1.7 (1.6)	1.23E‐03
Medical history, %									
Diabetes	11.6%	21.7%	0.0061	12.2%	30.0%	0.0019	11.0%	15.4%	0.3505
Hypertension	34.9%	47.8%	0.0151	32.5%	62.5%	0.0002	37.8%	36.5%	0.8662
CHF	1.5%	6.5%	0.0011	1.2%	7.5%	0.0042	1.7%	5.8%	0.0673
CVA	0.7%	0%	0.4340	0.5%	0%	0.6576	0.9%	0%	0.5009
Dementia	0.1%	0%	0.7271	0%	0%	—	0.3%	0%	0.6983
COPD	0.3%	0%	0.6214	0%	0%	—	0.6%	0%	0.5831
Cancer	0.1%	0%	0.7271	0.2%	0%	0.7542	0%	0%	—

### 2.2 Plasma Proteins and Metabolites Associated With Mortality

**FIGURE 1:**
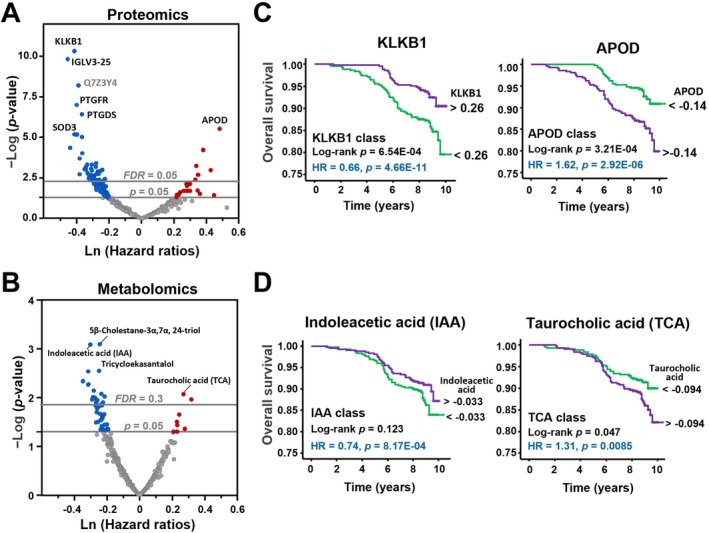
Association of plasma proteome and metabolome with all‐cause mortality. (A, B) Volcano plots showing associations between plasma proteins (A) and metabolites (B) with all‐cause mortality. Cox proportional hazards models were adjusted for age, sex, education, alcohol consumption, smoking status, hypertension, and diabetes. Horizontal gray lines indicate significance thresholds (*p* = 0.05; FDR = 0.05 for proteins, FDR = 0.3 for metabolites). See also Tables S1 and S2. (C, D) Kaplan–Meier survival curves for representative proteins (C; KLKB1 and APOD) and metabolites (D; indoleacetic acid and taurocholic acid), stratified by median levels (purple line: > median; green line: < median). *p*‐values are from log‐rank tests; hazard ratios (HR) and *p*‐values from adjusted Cox models are shown.

We examined associations between 532 metabolites and 516 proteins with all‐cause mortality using Cox proportional hazards models adjusted for age, sex, education, alcohol use, smoking status, hypertension, and diabetes. A total of 111 proteins and 55 metabolites demonstrated statistically significant associations with mortality at the nominal significance level (Figure 1A,B, Tables S1 and S2). Among the 111 proteins, 47 remained significant after correction for multiple testing (FDR < 5%). The proteins most strongly associated with mortality were KLKB1 and IGLV3‐25 (inverse associations, indicating lower mortality risk with higher levels) and APOD (positive association, indicating higher mortality risk with higher levels). Among metabolites, 5β‐cholestane‐3α,7α,24‐triol and indoleacetic acid showed the strongest inverse associations with mortality, while taurocholic acid demonstrated the strongest positive association. Overall, metabolite associations were weaker than protein associations; no metabolites survived FDR correction at 5%, though 27 met a more lenient threshold of FDR < 30%. Kaplan–Meier survival curves stratified at the median levels of KLKB1, APOD, and taurocholic acid showed clear separation over time (log‐rank *p* < 0.05; Figure 1C,D). Sensitivity analyses incorporating additional adjustment for congestive heart failure or replacing hypertension and diabetes with the Charlson Comorbidity Index showed high concordance in both effect sizes and statistical significance across all tested proteins and metabolites (Figure S1), indicating that these associations are robust to different approaches for accounting for comorbidity burden.

### 2.3 Tissue Origin of Mortality‐Associated Proteins

To identify the tissue origins of mortality‐associated proteins, we mapped the 79 significant proteins with FDR < 0.1 to human organ bulk RNA sequencing data from the GTEx project database using a previously described approach (Oh et al. 2023). To identify more subtle organ‐specific expression patterns, we modified the original enrichment criteria by reducing the fold‐change threshold from 4 to 1.5. Proteins were classified as ‘organ‐preferential’ when their expression in one organ exceeded that in all other organs by at least 1.5‐fold. Of the 79 mortality‐associated proteins, 36 (46%) were classified as organ‐preferential. The predominant tissue origins were liver (28%), immune system (25%), and brain (17%) (Figure S2), suggesting that hepatic, immune, and neurological aging processes play important roles in mortality risk.

### 2.4 Biological Functions and Pathways Associated With Mortality

**FIGURE 2:**
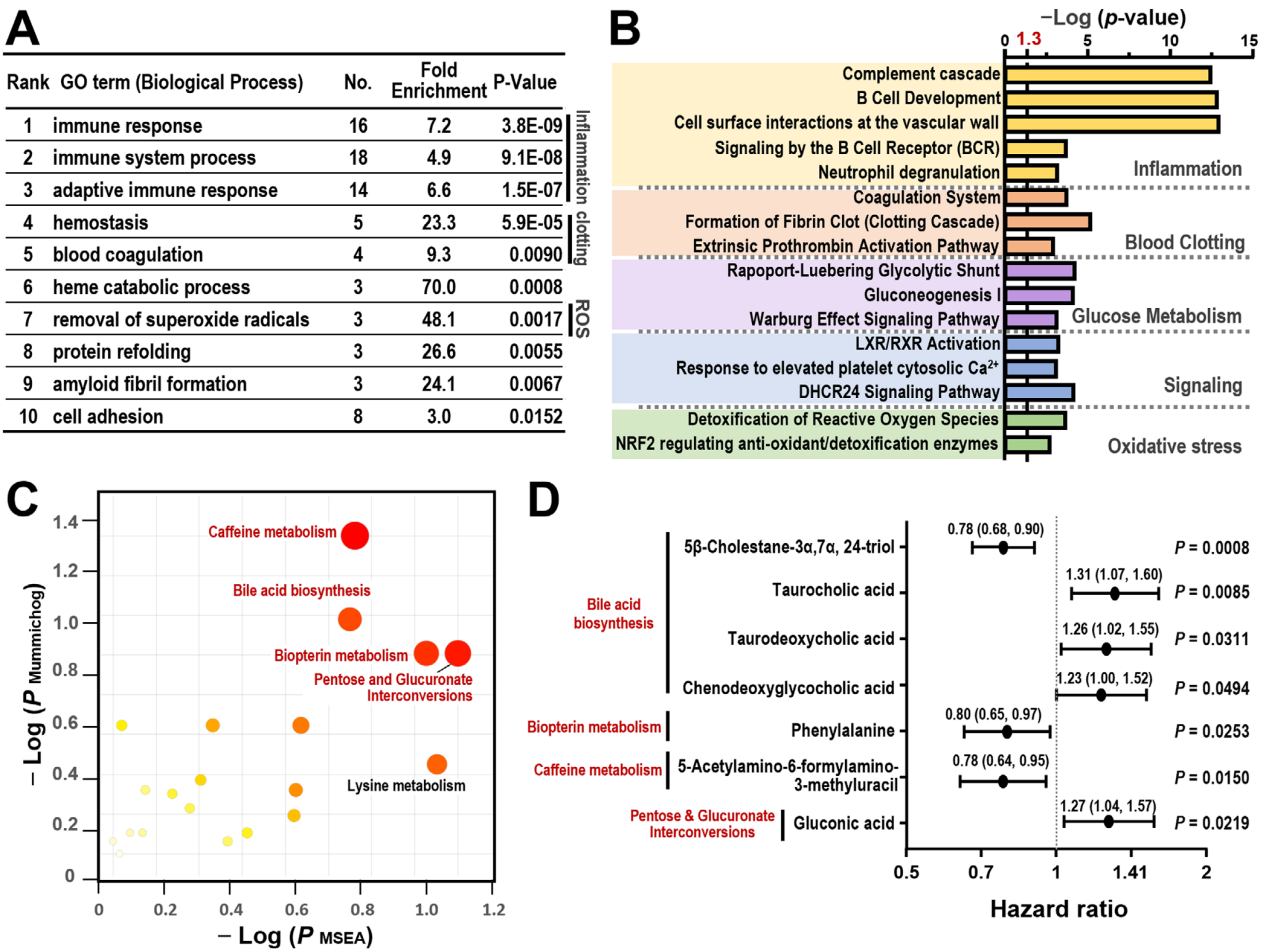
Biological pathway enrichment analysis of proteins and metabolites associated with all‐cause mortality. (A) Over‐represented Gene Ontology Biological Processes for 79 mortality‐associated proteins identified by multivariable‐adjusted Cox regression. The top 10 processes with a *p*‐value ≤ 0.015 are displayed. (B) Canonical pathway enrichment analysis of 79 mortality‐associated proteins using Ingenuity Pathway Analysis (IPA), grouped by functional categories: inflammation, blood clotting, glucose metabolism, signaling, and oxidative stress. (C) Pathway enrichment analysis of mortality‐associated metabolites using metabolite set enrichment analysis (MSEA) and Mummichog algorithms. Dot size represents the combined *p*‐value calculated by Fisher's method. Pathway names in red indicate a combined *p*‐value ≤ 0.1 (D) Metabolites within enriched metabolic pathways associated with all‐cause mortality. Hazard ratios and 95% confidence intervals were estimated using multivariable‐adjusted Cox proportional hazards models.

Gene Ontology (GO) enrichment analysis of the 79 mortality‐associated proteins revealed significant enrichment for inflammatory processes, blood coagulation, removal of superoxide radicals, and protein refolding (Figure 2A). Ingenuity Pathway Analysis (IPA) identified the most significantly enriched canonical pathways as inflammatory response and coagulation system, followed by glucose metabolism, signaling, and oxidative stress pathways (Figure 2B). These biological processes were closely linked to metabolic health and aging.

For the metabolomics dataset, we conducted metabolite set enrichment analysis (MSEA) and applied the Mummichog algorithm to identify pathway‐level associations with mortality. Four metabolic pathways demonstrated significant associations (combined *p* < 0.1): (1) caffeine metabolism; (2) pentose and glucuronate interconversions; (3) biopterin metabolism; and (4) bile acid biosynthesis (Figure 2C). At the metabolite level, 5‐acetylamino‐6‐formylamino‐3‐methyluracil, mapped to the caffeine metabolism pathway, showed an inverse association with mortality risk (HR = 0.78). Within the bile acid biosynthesis pathway, an upstream intermediate, 5β‐cholestane‐3α,7α,24‐triol, was inversely associated with mortality risk (HR = 0.78), whereas several downstream conjugated bile acids—including glycochenodeoxycholic acid, taurocholic acid, and taurodeoxycholic acid—were positively associated with mortality (HR = 1.23–1.31), as shown in Figure 2D.

### 2.5 Plasma Metabolomic and Proteomic Profiles Associated With Longevity

We further investigated associations between plasma metabolites and proteins with longevity, defined as survival to age ≥ 85 years for women and ≥ 80 years for men based on the life expectancy in Taiwan. After a mean follow‐up of 8.5 years, 119 participants (14.0%) achieved longevity during the study period. Demographic and clinical characteristics of the study population stratified by longevity status were presented in Table S3. Among baseline comorbidities, only the absence of congestive heart failure was significantly associated with longevity (*p* = 0.0392).

**FIGURE 3:**
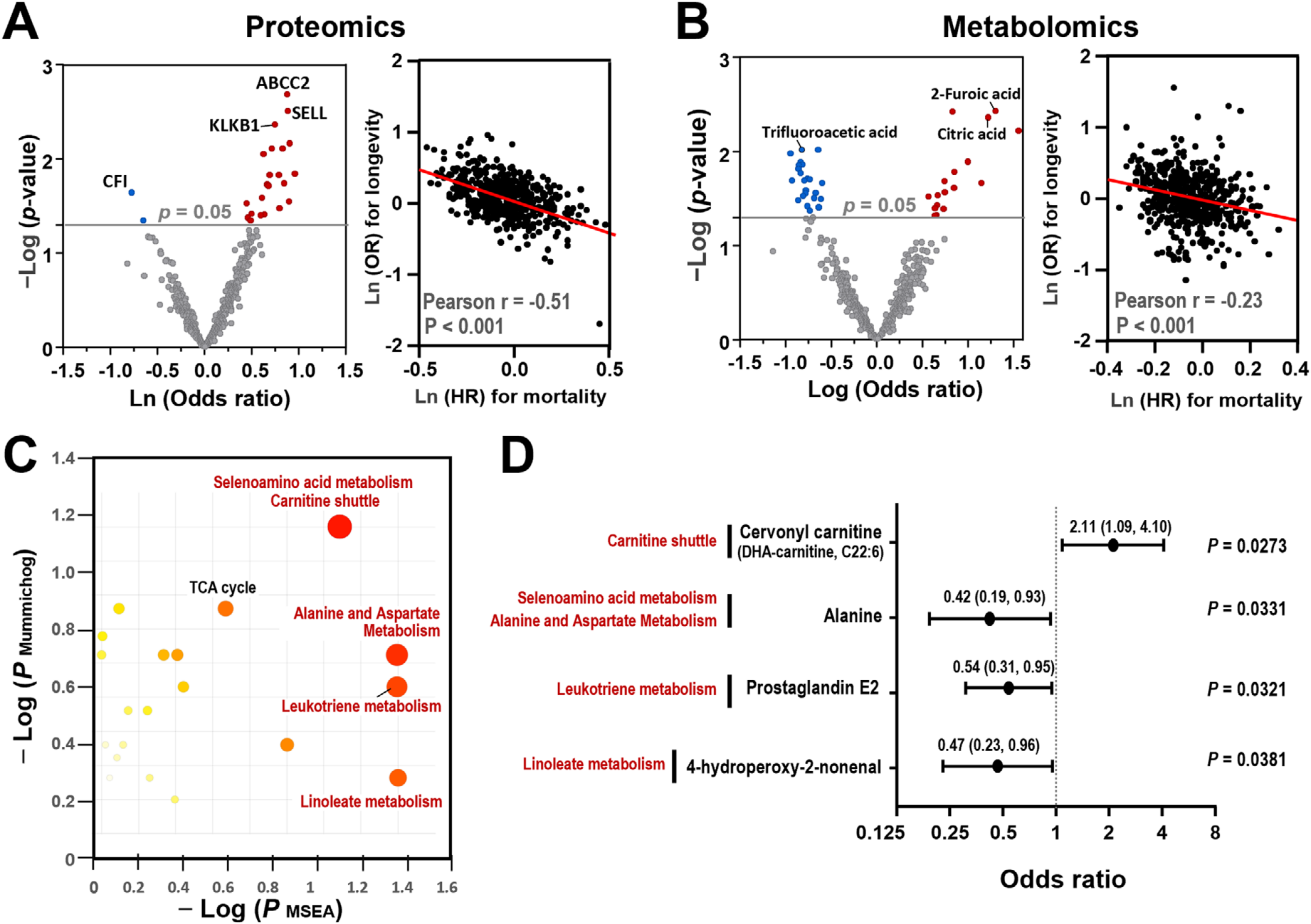
Proteomic and metabolomic signatures associated with longevity. (A) Volcano plot of plasma protein associations with longevity from logistic regression. Scatter plot demonstrates significant inverse correlation (*r* = −0.51, *p* < 0.001) between ln(OR) for longevity and ln(HR) for all‐cause mortality. (B) Volcano plot of plasma metabolite associations with longevity from logistic regression. Scatter plot shows modest inverse correlation (*r* = −0.23, *p* < 0.001) between ln(OR) for longevity and ln(HR) for all‐cause mortality. (C) Pathway enrichment analysis of longevity‐associated metabolites using MSEA and Mummichog algorithms. Dot size represents combined *p*‐value by Fisher's method. Pathway names in red indicate a combined *p*‐value ≤ 0.1. (D) Metabolites within enriched metabolic pathways associated with longevity. Odds ratios and 95% confidence intervals were estimated using multivariable‐adjusted logistic regression models.

Using logistic regression models adjusted for age, sex, education, alcohol use, smoking status, hypertension, and diabetes, we identified 24 proteins and 40 metabolites nominally associated with longevity (*p* < 0.05; Figure 3A,B). However, none of these associations remained significant after correction for multiple testing (FDR < 0.05). When comparing effect directions between mortality and longevity analyses, we observed a stronger inverse correlation for proteins (Pearson *r* = −0.51) than for metabolites (Pearson *r* = −0.23). By resampling‐based analysis, the inverse correlation observed in the proteomic profile was significantly stronger than expected by chance (empirical *p* = 0.003; Figure S3A), supporting a coordinated directional shift and a system‐level pattern. In contrast, no significant deviation from chance was observed for metabolomics (empirical *p* = 0.134; Figure S3B). Of note, KLKB1 emerged as one of the most significantly associated proteins with longevity, with the effect direction opposite that observed for mortality (i.e., higher levels associated with both lower mortality and higher odds of longevity). This bidirectional association suggested that KLKB1 may serve as an important biomarker or regulator of lifespan.

Pathway enrichment analysis of longevity‐associated metabolites identified five significantly enriched pathways (combined *p* ≤ 0.1): carnitine shuttle, selenoamino acid metabolism, alanine and aspartate metabolism, leukotriene metabolism and linoleate metabolism (Figure 3C,D). Within the carnitine shuttle pathway, cervonyl carnitine (DHA‐carnitine) was the key annotated metabolite with a positive association with longevity (OR = 2.11, *p* = 0.027). For linoleate metabolism, 4‐hydroperoxy‐2‐nonenal (4‐HpNE), a lipid peroxidation product, was identified as the primary metabolite responsible for the association. Higher levels of 4‐HpNE were associated with lower odds of achieving longevity (OR = 0.47, *p* = 0.038), consistent with the detrimental effects of oxidative stress on lifespan, although the association between 4‐HpNE and all‐cause mortality did not reach statistical significance.

### 2.6 Development of Proteomic and Metabolomic Scores for Predicting Mortality

**FIGURE 4:**
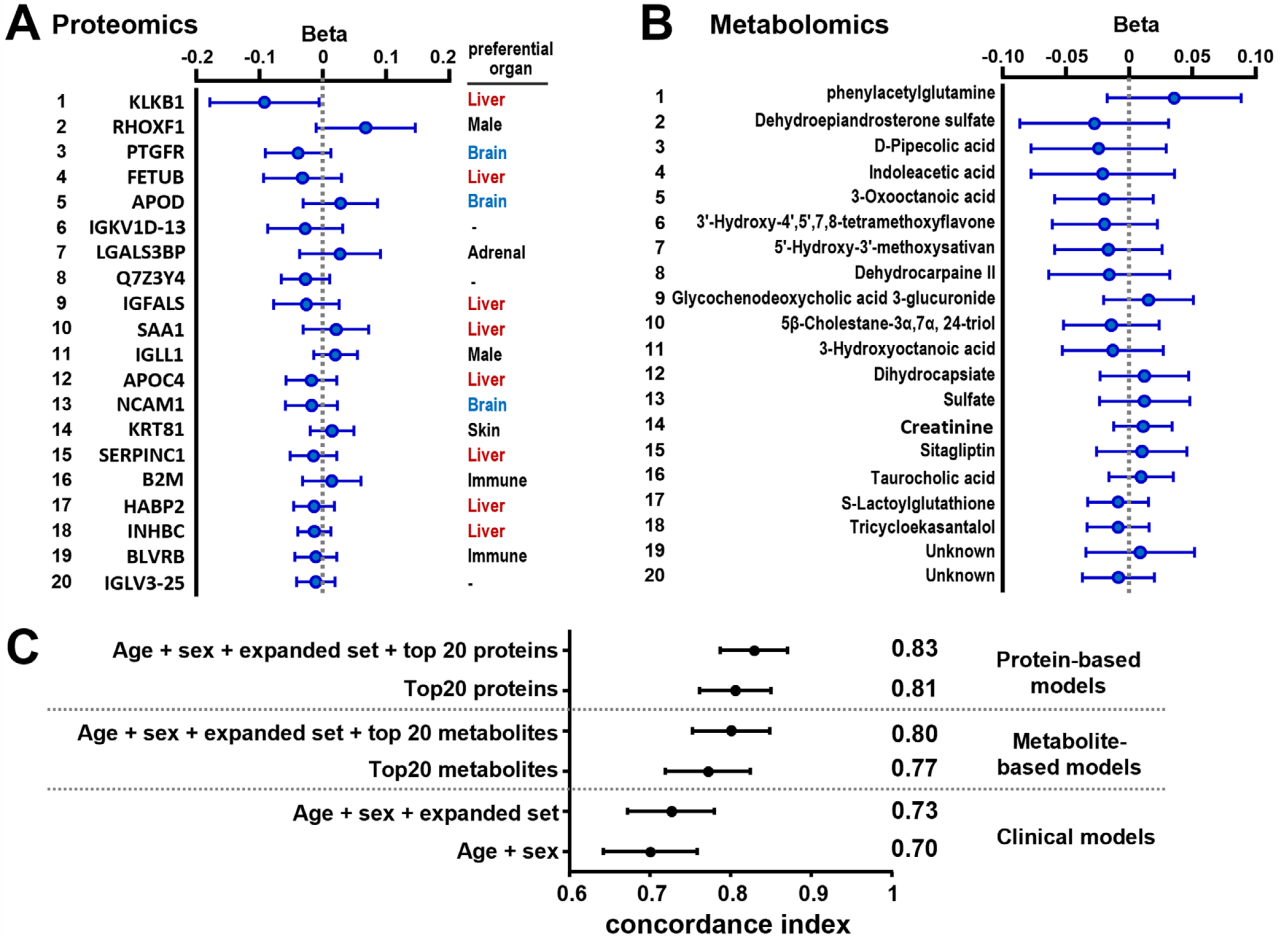
Construction of ProteinScore and MetaboliteScore for mortality prediction using Cox proportional hazards elastic net regression. (A, B) Forest plots displaying the top 20 plasma proteins (A) and metabolites (B) with the highest mean absolute coefficients associated with all‐cause mortality risk. (C) Performance comparison of Cox regression models incorporating the top 20 proteomic or metabolomic biomarkers, with or without clinical covariates (age, sex, education, alcohol consumption, smoking status, hypertension, and diabetes). Proteomic biomarkers demonstrated superior prognostic accuracy compared to metabolomic biomarkers and clinical variables alone.

To develop parsimonious prognostic models, we applied elastic net regression to select proteins and metabolites most predictive of mortality. Across 50 randomized iterations, the top 20 proteomic and top 20 metabolomic features were selected based on mean absolute coefficient magnitudes (Figure 4A,B). The five proteins with the highest predictive weights were KLKB1, PHOXF1, PTGFR, FETUB, and APOD. The five most predictive metabolites were phenylacetylglutamine, dehydroepiandrosterone sulfate (DHEA‐S), pipecolic acid, indoleacetic acid, and 3‐oxooctanoic acid.

We evaluated model performance using the concordance index (*C*‐index) for three modeling approaches: (1) protein‐based score alone (ProteinScore; derived from top 20 proteins), (2) metabolite‐based score alone (MetaboliteScore; derived from top 20 metabolites), and (3) each score combined with clinical covariates (age, sex, education, smoking status, alcohol use, hypertension, and diabetes history; Figure 4C). The ProteinScore achieved superior discrimination (*C*‐index = 0.81) compared to clinical covariates alone (*C*‐index = 0.73), and the MetaboliteScore (*C*‐index = 0.77) also outperformed clinical covariates. When combining omics scores with clinical features, model performance improved further, with *C*‐indices increasing to 0.83 for the protein score model and 0.80 for the metabolite score model.

In sensitivity analyses using only the top 10 proteins or metabolites, both scores showed reduced *C*‐indices compared to the top 20 features, with or without clinical features (Figure S4). Notably, the MetaboliteScore derived from the top 10 metabolites performed comparably to clinical covariates alone. Across all analyses, the combined ProteinScore with clinical features demonstrated the best predictive performance, whether using the top 10 or top 20 proteomic features.

### 2.7 Validation in an Independent Cohort

**FIGURE 5:**
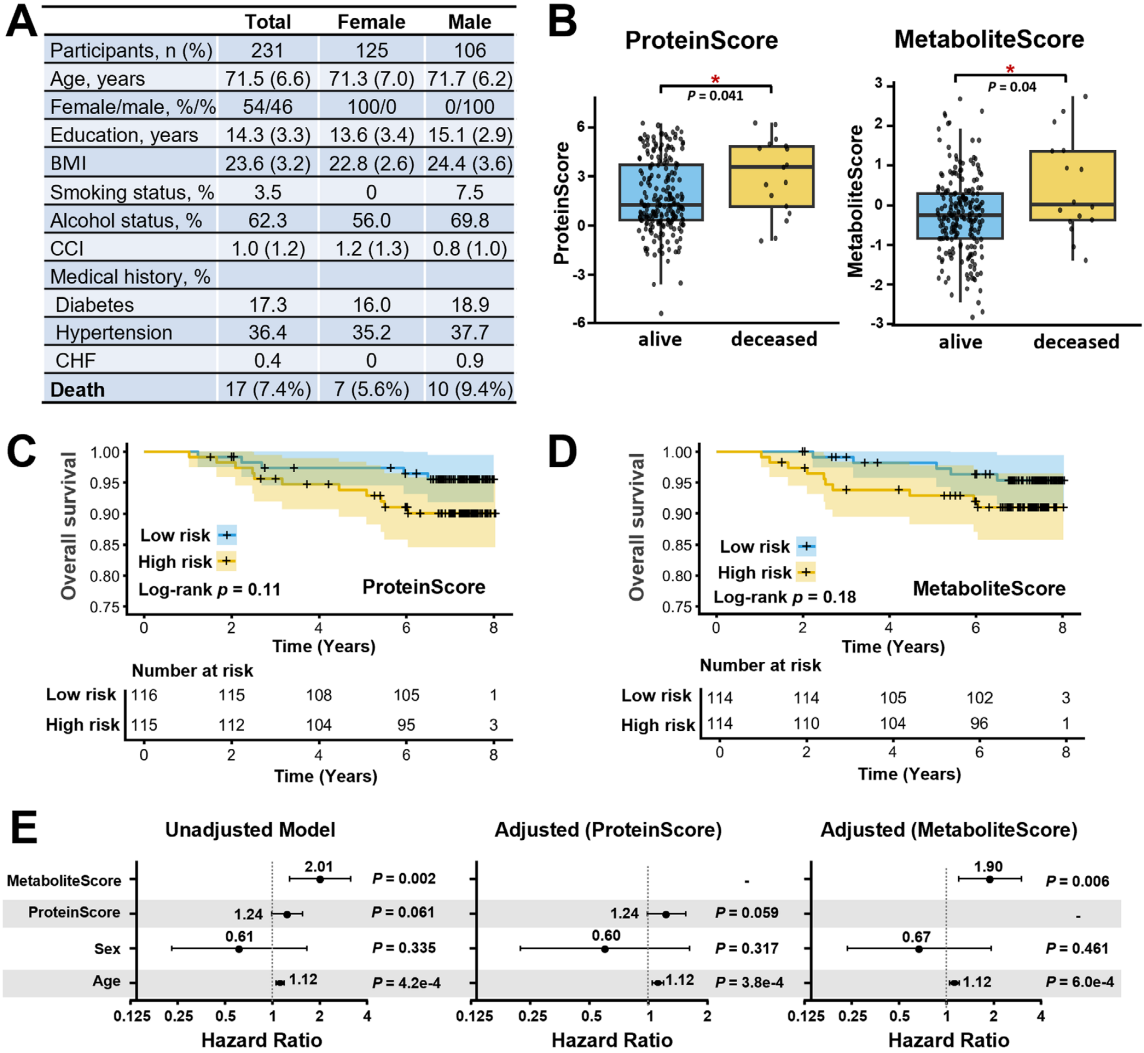
Validation of ProteinScore and MetaboliteScore in the LAST cohort. (A) Demographic and clinical characteristics of the validation cohort (*n* = 231). (B) Distribution of ProteinScore and MetaboliteScore values, calculated from the top 20 proteomic or metabolomic predictors, comparing deceased and surviving participants. Box plots display median, interquartile range, and individual data points. (C) Kaplan–Meier survival curves stratified by median ProteinScore. High ProteinScore (above median) was associated with poorer survival compared to low ProteinScore (*p* = 0.11, log‐rank test). (D) Kaplan–Meier survival curves stratified by median MetaboliteScore. High MetaboliteScore (above median) was associated with poorer survival compared to low MetaboliteScore (*p* = 0.18, log‐rank test). (E) Forest plots of hazard ratios (HR) for all‐cause mortality. The left panel shows unadjusted ProteinScore, MetaboliteScore, and age/sex models. The middle and right panels show models adjusted for age and sex. HRs are shown with 95% confidence intervals and *p*‐values.

We next evaluated the omics‐based mortality risk scores in an independent cohort from the Longitudinal Aging Study of Taipei (LAST). During a median follow‐up of 7.3 years, 17 of 231 participants (7.4%) died (Figure 5A). For both ProteinScore and MetaboliteScore, deceased participants had significantly higher risk scores than survivors (Figure 5B), indicating consistent directional associations with mortality. However, Kaplan–Meier analyses using median‐based stratification did not show statistically significant survival differences (log‐rank *p* = 0.11 for ProteinScore and *p* = 0.18 for MetaboliteScore; Figure 5C,D), likely reflecting the limited number of events in this cohort. In Cox regression models, the MetaboliteScore was significantly associated with mortality (HR = 1.9, *p* = 0.006), whereas the ProteinScore showed a borderline association (HR = 1.2, *p* = 0.059). Adjustment for age and sex did not alter these estimates, suggesting that the observed associations were not fully explained by these covariates (Figure 5E).

## 3 Discussion

Our study leveraged untargeted proteomics and metabolomics to identify circulating biomarkers associated with mortality risk in a Taiwanese aging cohort (I‐Lan Longitudinal Aging Study, ILAS). We identified mortality‐associated signatures comprising 20 proteins and 20 metabolites that demonstrated superior discrimination of high‐risk individuals compared to traditional clinical characteristics. Notably, the mortality risk score models derived from elastic net regression did not incorporate age, sex, or other clinical covariates as predictors. This methodological difference accounted for the inclusion of certain biomarkers in the elastic net model that did not achieve statistical significance (*p* < 0.05) in age‐ and sex‐adjusted Cox regression. For instance, phenylacetylglutamine and dehydroepiandrosterone sulfate (DHEA‐S) were selected as prominent predictors in the elastic net model despite lacking individual statistical significance in adjusted analyses. This observation emphasized that elastic net prioritized variables based on their collective predictive capacity, potentially capturing age‐related and other complex biological interactions. These findings underscored a fundamental principle of modern biological aging research: molecular biomarker‐based risk stratification surpassed traditional clinical phenotypic assessments in mortality prediction, reflecting the paradigm shift from chronological age and observable clinical characteristics toward molecular signatures that capture the underlying biological processes driving aging and age‐related outcomes.

The mortality‐associated proteomic and metabolomic signatures identified in this study provided molecular evidence supporting the interconnected nature of aging hallmarks as conceptualized in the expanded framework by López‐Otín and colleagues (Lopez‐Otin et al. 2023). The enrichment of mortality‐associated proteins in inflammatory response pathways is consistent with inflammaging, characterized by sustained low‐grade inflammation that drives age‐related pathological processes. Metabolites linked to microbiota–host interactions, particularly phenylacetylglutamine and pipecolic acid, were consistent with a potential contribution of age‐related dysbiosis to mortality risk. The enrichment of proteins involved in reactive oxygen species clearance and oxidative stress pathways implicated mitochondrial dysfunction and loss of proteostasis, primary hallmarks reflecting accumulated cellular damage and impaired protein quality control (Kennedy et al. 2014). Notably, apolipoprotein D (APOD), the protein most strongly associated with mortality, represented a compensatory neuroprotective response to cellular stress and oxidative damage, exemplifying how altered intercellular communication manifested as both adaptive and maladaptive signaling alterations during aging (Annema et al. 2022). The convergence of proteins from hepatic, immune, and neurological systems with metabolites spanning bile acid metabolism and lipid peroxidation illustrated how mortality risk emerged from the cumulative dysregulation of interconnected molecular networks rather than from isolated pathway perturbations, validating the integrative, cross‐hallmark perspective central to contemporary geroscience research (Moqri et al. 2023).

Historically, the liver was considered remarkably resilient to aging, with routine liver function tests remaining largely stable across the lifespan despite early observations of age‐related pseudocapillarization, reduced hepatic blood flow, and morphological changes in sinusoidal endothelial cells (Le Couteur et al. 2001; McLean et al. 2003). However, recent studies using organ‐specific proteomic aging signatures have increasingly implicated the liver as an important contributor to mortality risk and age‐related diseases (Oh et al. 2023, 2025; Y. Wang et al. 2026; Zhao et al. 2025). Our findings align with and extend this evolving perspective. While recent chronologically trained organ‐aging clocks frequently highlight the brain as a prominent component (Oh et al. 2025; Q. Wang et al. 2025; Zhao et al. 2025), our analysis employed a mortality‐driven objective to identify molecular features more directly associated with survival outcomes. Under this framework, the liver emerged as the predominant hub. Among the 111 mortality‐associated proteins identified, approximately 47% demonstrated organ‐preferential expression. The liver accounted for 28% of these tissue‐specific signatures—the largest contribution of any single organ system, exceeding both the immune system (25%) and brain (17%). This hepatic predominance extended to our elastic net mortality prediction model, where 8 of the top 20 proteomic predictors (40%) were liver‐derived, including KLKB1, FETUB, IGFALS, SAA1, APOC4, SERPINC1, HABP2, and INHBC. Several proteins identified in our mortality prediction model overlapped with previously reported hepatic aging proteomic signatures. For example, FETUB, KLKB1, and SERPINC1 have also been included in independent liver‐aging proteomic models (Oh et al. 2023, 2025; Y. Wang et al. 2026). The convergence of these specific proteins across different modeling strategies—predicting chronological age versus predicting mortality—validates their roles as critical nodes linking hepatic functional decline to systemic survival. These proteins orchestrated critical hepatic functions spanning coagulation cascades, lipid metabolism, insulin signaling, and acute‐phase inflammatory responses—pathways that became progressively dysregulated during aging (Lopez‐Otin et al. 2023).

Complementing these proteomic findings, the hepatocyte‐synthesized taurocholic acid demonstrated the strongest positive association with mortality among all metabolites examined, with elevated levels linked to progression of liver fibrosis through activation of hepatic stellate cells via toll‐like receptor 4 upregulation (Liu et al. 2018). These findings established that hepatic aging represented not merely an organ‐specific process but a systems‐level driver of biological aging and mortality risk. The liver emerged as a potentially modifiable therapeutic target for longevity interventions, with hepatoprotective strategies—including management of metabolic dysfunction‐associated steatotic liver disease, mitigation of hepatic inflammation, and preservation of sinusoidal endothelial function—warranting prioritization in comprehensive aging intervention programs (Gan et al. 2011).

Two metabolites—pipecolic acid and 3‐hydroxyoctanoic acid—exhibited significant associations with both mortality and longevity in opposite directions, representing novel findings that warranted mechanistic investigation. Pipecolic acid, an intermediate of lysine catabolism oxidized in peroxisomes, accumulated in peroxisome biogenesis disorders and has been identified as a component of biomarker panels for hepatocellular carcinoma risk stratification in persons with metabolic syndrome (Cao et al. 2021). Our observation that lower pipecolic acid levels were associated with increased mortality while higher levels were associated with longevity suggested a previously unrecognized role in healthy aging. Similarly, 3‐hydroxyoctanoic acid, a β‐oxidation intermediate that exerts anti‐lipolytic activity through hydroxycarboxylic acid receptor 3 (HCAR3) signaling (Ahmed 2011), demonstrated parallel but opposite associations with survival outcomes. The absence of existing literature connecting this metabolite to mortality outcomes identified it as a priority target for mechanistic studies to elucidate its role in longevity pathways.

In the discovery cohort, ProteinScore showed stronger mortality discrimination than MetaboliteScore and clinical characteristics alone. However, the predictive performance of both omics‐derived scores was attenuated in the external validation cohort. This attenuation may reflect inter‐cohort heterogeneity, including differences in demographic structure, education level, environmental exposure, and lifestyle factors between ILAS and LAST. The metabolomic score showed stronger hazard associations in Cox models, whereas the proteomic score demonstrated slightly better group separation in Kaplan–Meier analyses (Figure 5C–E). These observations suggest that different evaluation frameworks may capture distinct aspects of prognostic performance, including continuous risk association versus population‐level risk stratification. The partially discordant findings across evaluation metrics suggest that proteomic and metabolomic signatures may capture complementary biological dimensions of mortality risk.

Proteomic signatures may reflect cumulative physiological dysregulation, immune–hepatic remodeling, and chronic systemic states, whereas metabolomic signatures may capture dynamic metabolic responses and short‐term influences such as dietary intake, medication exposure, and fasting status. Future studies incorporating genomic, epigenomic, transcriptomic, proteomic, metabolomic, and neuroimaging data may further improve the robustness and biological interpretability of biological aging assessment by capturing the multidimensional nature of aging trajectories.

This study has several limitations that warrant consideration. First, we employed all‐cause mortality as the primary outcome and were unable to examine cause‐specific mortality, which may involve distinct biological mechanisms. Second, the external validation cohort had a limited sample size and relatively few mortality events, which may have reduced statistical power and contributed to the inconsistent performance across evaluation metrics. Nevertheless, the proteomic score retained directionally consistent associations with mortality‐related outcomes, suggesting that the identified hepatic proteomic signatures may still capture biologically relevant aspects of aging and mortality risk. Third, the single‐timepoint measurement of proteomic and metabolomic profiles limited our ability to assess temporal dynamics of biomarker changes during aging. Longitudinal studies with repeated multi‐omics measurements would help clarify whether the observed associations reflect causal pathways, compensatory responses, or age‐related physiological adaptations. Further mechanistic studies are needed to determine the biological roles of the identified biomarkers in aging and mortality.

In summary, the present study identified liver‐ and immune‐enriched molecular signatures associated with mortality risk in aging populations. Although predictive performance was attenuated in the external validation cohort, the proteomic and metabolomic signatures retained associations with mortality‐related outcomes and highlighted distinct molecular features of aging and mortality risk. In particular, hepatic coagulation‐related proteins and bile acid–related metabolites, together with molecular features potentially linked to microbiota–host interactions, emerged as biologically relevant candidates underlying aging‐related mortality. Collectively, these findings support the potential of integrated plasma multi‐omics profiling to refine mortality risk stratification and advance the molecular understanding of aging in older adults.

## 4 Methods

### 4.1 Study Design and Population

The I‐Lan Longitudinal Aging Study (ILAS) is a prospective cohort study investigating relationships among frailty, sarcopenia, and cognitive decline in aging adults. The study design, recruitment procedures, and data collection methods have been described previously (Lee et al. 2022). Community‐dwelling adults aged ≥ 50 years were recruited. Exclusion criteria included inability to communicate effectively with research staff, limited life expectancy due to severe illness, residence in long‐term care facilities, or disabilities precluding assessment completion. Participants were followed for mortality through 2022 via telephone contact with participants or next of kin.

At baseline, trained personnel collected data on education level, smoking status, alcohol consumption, and comorbidities (diabetes and hypertension) via self‐reported questionnaires and measured anthropometric variables and grip strength according to standardized protocols. The final analytical sample comprised 848 participants for prospective mortality analysis using Cox proportional hazards regression. For longevity analysis, a sub‐cohort was further defined using sex‐specific longevity thresholds (≥ 85 years for females and ≥ 80 years for males). Participants who remained alive at the end of follow‐up but had not yet reached these thresholds were excluded because their longevity status could not be definitively determined. The final longevity analysis included 183 participants and was performed using logistic regression. Validation of the mortality risk score utilized 231 participants from the first wave of the Longitudinal Aging Study of Taipei (LAST; 2016–2019) who completed proteomic and metabolomic assessments.

### 4.2 Proteomics Analysis

Plasma samples were collected from fasting blood, processed, and analyzed by nanoLC‐nanoESI‐MS/MS, as previously described (Huang et al. 2025). Briefly, proteins were trypsin‐digested using the SMART Digest kit (Thermo Fisher Scientific) and analyzed by the LTQ Orbitrap Elite mass spectrometer. Protein identification was performed in Proteome Discoverer (v2.4) by database searching against the UniProt human database, while protein quantification was carried out using MS1‐based label‐free quantification with normalization to total peptide intensity. Proteins detected in ≥ 50% of samples (516 proteins) were retained for further analysis.

### 4.3 Metabolomics Analysis

Plasma metabolomics was performed as previously described (Huang et al. 2025). Plasma samples were deproteinized, spiked with isotope‐labeled internal standards (lysine‐^13^C6 and stearic acid‐^13^C18, 1 ppm each), and analyzed by LC–MS using a Waters Xevo G2‐S Qtof MS coupled to an Acquity UPLC system. A 9‐min gradient elution (1%–100% acetonitrile with 0.1% ammonium hydroxide) was employed. Data were acquired in negative ion MSE mode (50–1200 m/z) with continuous mass calibration using leucine enkephalin. Chemical features were detected and quantified using Progenesis QI software and normalized against pooled quality control samples to reduce batch effects. Metabolites were annotated using the Human Metabolome Database (HMDB), and features with > 20% missing values were excluded, yielding 532 metabolites for analysis.

### 4.4 Univariate Survival and Longevity Analyses

All metabolomic and proteomic data were log‐transformed and standardized to *z*‐scores prior to analysis. The associations between individual metabolites or proteins and all‐cause mortality were evaluated using Cox proportional hazards regression models adjusted for age, sex, education level (years), smoking status, alcohol consumption, and history of hypertension and diabetes. Cox regression analysis was performed using MATLAB (MathWorks, Natick, MA, USA). Person‐time for each participant was calculated from the date of blood collection until either the date of death or the end of follow‐up (April 2022). The resulting *p* values were adjusted using the Benjamini–Hochberg method.

Kaplan–Meier survival curves were generated using SPSS (IBM Corp.). Participants were stratified into two groups based on the median value of each metabolite or protein (above or below median), and survival differences between groups were assessed using log‐rank tests. The associations between individual metabolites or proteins and longevity were evaluated using logistic regression models with the same covariate adjustments as the mortality analyses. Longevity was defined as survival to age 85 years for females or 80 years for males. Participants who were alive but had not yet reached the age threshold for longevity at the end of follow‐up were excluded from the longevity analysis. A total of 848 participants were included in the Cox regression analysis for mortality, and 183 participants were included in the logistic regression analysis for longevity.

### 4.5 Development and External Validation of Omics‐Derived Mortality Risk Scores

Proteomic‐ and metabolomic‐derived mortality risk scores were developed separately using elastic‐net penalized Cox proportional hazards regression in R version 4.4 (glmnet and survival packages). In the discovery cohort, an iterative feature selection procedure consisting of 50 randomized iterations was performed. For each iteration, participants were randomly divided into training and testing sets at a 1:1 ratio. To address class imbalance, non‐event participants in the training set were randomly undersampled to achieve an approximately 1:3 event‐to‐non‐event ratio. Elastic‐net Cox models were trained using threefold cross‐validation, and model performance in the testing set was evaluated using Harrell's concordance index. Features with non‐zero coefficients were recorded across iterations, and the final ProteinScore and MetaboliteScore were constructed using the top 20 features ranked by mean absolute coefficient values across all iterations. Selected omics features were standardized in the discovery cohort, and Cox proportional hazards models were fitted to generate omics‐derived mortality risk scores as linear predictors.

Three Cox proportional hazards models were further evaluated in the discovery cohort: (1) a clinical model including age, sex, education, smoking status, alcohol consumption, hypertension, and diabetes; (2) an omics‐only model including the omics‐derived mortality risk score; and (3) a combined model including both clinical covariates and the omics‐derived score. For external validation, the same features in the LAST cohort were standardized using the mean and standard deviation derived from the discovery cohort, and mortality risk scores were calculated using the discovery cohort‐derived Cox coefficients. In the validation cohort, score distributions were compared between survivors and non‐survivors using the Wilcoxon rank‐sum test. Kaplan–Meier analysis and Cox proportional hazards models adjusted for age and sex were further applied to evaluate associations between omics‐derived scores and mortality risk.

### 4.6 Statistical and Pathway Enrichment Analyses

Demographic and clinical characteristics are presented as mean (standard deviation, SD) for continuous variables and as number (percentage) for categorical variables. Between‐group comparisons were performed using Student's *t*‐test for continuous variables and chi‐square test for categorical variables. Gene Ontology (GO) enrichment analyses for proteomic data were conducted using DAVID Bioinformatics Resources version 6.8 (http://david.abcc.ncifcrf.gov/). Canonical pathways associated with mortality were identified using Ingenuity Pathway Analysis (IPA; QIAGEN). GO enrichment and IPA canonical pathway analyses for proteomic data were performed using mortality‐associated proteins passing false discovery rate correction (FDR < 0.1).

For metabolomic data, pathway enrichment analyses were performed using metabolite set enrichment analysis (MSEA) and the Mummichog algorithm implemented in MetaboAnalyst 6.0 (https://www.metaboanalyst.ca/). In MSEA, metabolites were ranked according to *Z*‐statistics (β/SE). Mummichog analysis was performed using metabolites with nominal significance (*p* < 0.05) (Li et al. 2013). For selected enriched pathways, matched metabolites and their corresponding hazard ratios, 95% confidence intervals, and *p*‐values were summarized. Volcano plots, forest plots, dot plots, and correlation scatter plots were generated using GraphPad Prism version 8.4.

## Author Contributions

Li‐Ning Peng, Fei‐Yuan Hsiao, and Liang‐Kung Chen conceived and designed the study. Wei‐Ju Lee, Pei‐Lin Lee, and Yi‐Long Huang developed the methodology. Yi‐Long Huang and Pei‐Lin Lee performed Cox and elastic net regression analyses. Yi‐Long Huang drafted the initial manuscript. Liang‐Kung Chen edited the manuscript and coordinated project administration including data access. Fei‐Yuan Hsiao, Li‐Ning Peng, and Liang‐Kung Chen supervised the study. All authors reviewed and approved the final version of the manuscript.

## Funding

This work was supported by the Interdisciplinary Research Center for Healthy Longevity of National Yang Ming Chiao Tung University from the Featured Areas Research Center Program within the framework of the Higher Education Sprout Project by the Ministry of Education in Taiwan. This work was also supported by the National Science and Technology Council, Taiwan (NSTC 114‐2321‐B‐A49‐012).

## Ethics Statement

All ILAS and LAST participants provided written informed consent administered by research nurses. The study protocol was approved by the Institutional Review Board of National Yang Ming University (No. YM103008 for ILAS; YM104121F‐3 for LAST) and conducted in accordance with the Declaration of Helsinki.

## Conflicts of Interest

The authors declare no conflicts of interest.

## Supporting information


**Figure S1:** Sensitivity analysis of comorbidity adjustment in Cox models across all tested proteins and metabolites.
**Figure S2:** Pie chart showing the distribution of organ‐preferential plasma proteins associated with all‐cause mortality.
**Figure S3:** Permutation testing of correlations between mortality‐ and longevity‐associated plasma biomarker effect sizes.
**Figure S4:** Performance comparison of Cox regression models incorporating the top 10 proteomic or metabolomic biomarkers.

**Table S1:** Association of plasma proteins with all‐cause mortality among participants in the ILAS1.

**Table S2:** Association of plasma metabolites with all‐cause mortality among participants in the ILAS1.

**Table S3:** Baseline characteristics of participants from the I‐Lan Longitudinal Aging Study (ILAS) included in the longevity analysis.

## Data Availability

Deidentified participant data are available from the corresponding author upon reasonable request.
