# Supplementary material for: Liver‐ and Immune‐Enriched Molecular Signatures Associated With Mortality in Older Adults
Source: Aging Cell. 2026 Jul 15;25(7):e70621. doi: 10.1111/acel.70621 (PMC13372077; doi:10.1111/acel.70621)
Supplement: Supplementary file 1 — Figure S1: Sensitivity analysis of comorbidity adjustment in Cox models across all tested proteins and metabolites. Figure S2: Pie chart showing the distribution of organ‐preferential plasma proteins associated with all‐cause mortality. Figure S3: Permutation testing of correlations between mortality‐ and longevity‐associated plasma biomarker effect sizes. Figure S4: Performance comparison of Cox regression models incorporating the top 10 proteomic or metabolomic biomarkers. [file ACEL-25-e70621-s002.pdf]

Figure S1

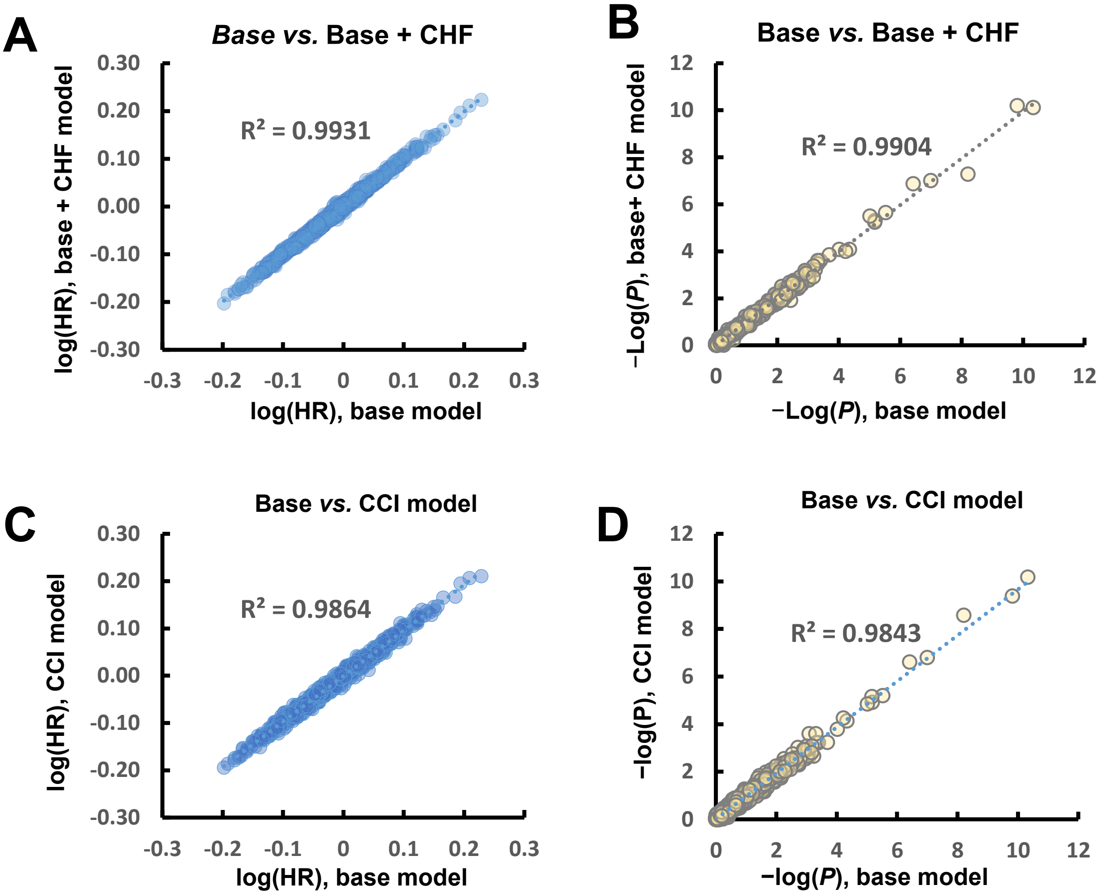

Figure S1. Sensitivity analysis of comorbidity adjustment in Cox models across all tested proteins and metabolites.

(A–B) Concordance of log(HR) and −log(*P*-value) between the base model and the base model additionally adjusted for congestive heart failure (CHF). **(C–D)** Concordance between the base model and the Charlson Comorbidity Index (CCI) model, in which hypertension and diabetes were replaced by CCI. Analyses included 516 proteins and 532 metabolites. The base model included age, sex, education, smoking status, alcohol use, hypertension, and diabetes. R^2 values are indicated in each panel.

Figure S2

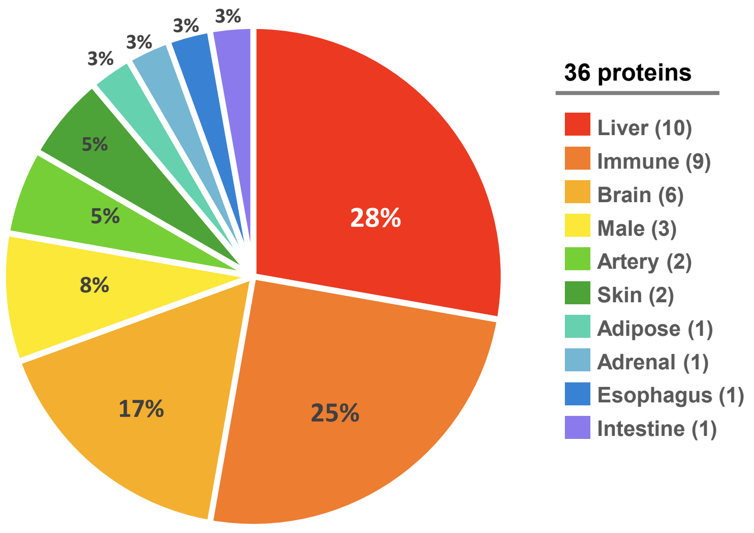

Figure S2. Pie chart showing the distribution of organ-preferential plasma proteins associated with all-cause mortality. Among the 79 plasma proteins identified at FDR < 0.1 in Cox regression analyses, 36 proteins were classified as organ-preferential if their expression level in one organ was at least 1.5-fold higher than that in any other organ. Numbers in parentheses indicate the number of proteins assigned to each organ category.

Figure S3

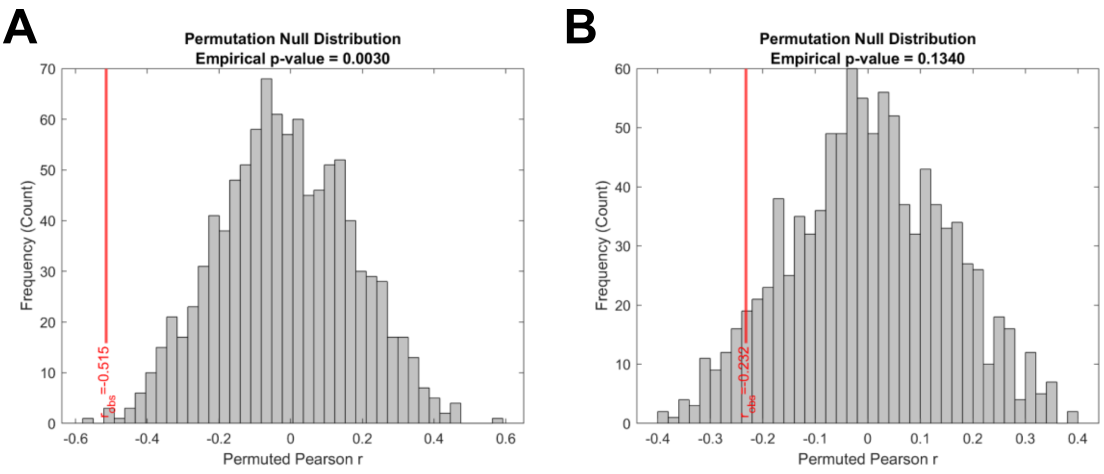

Figure S3. Permutation test validating the correlation between mortality-associated and longevity-associated plasma biomarker effect sizes.
(A) Null distribution of Pearson correlation coefficients (r) between log hazard ratios (log HR) from Cox proportional hazards regression and log odds ratios (log OR) from logistic regression across 516 plasma proteins, derived from 1,000 permutations of longevity labels. The red dashed vertical line indicates the observed r = −0.51. The empirical permutation *p*-value was 0.003. **(B)** Null distribution of Pearson correlation coefficients (r) between log HR and log OR across 532 plasma metabolites, derived from 1,000 permutations of longevity labels. The red dashed vertical line indicates the observed r = −0.23. The empirical permutation *p*-value was 0.134.

Figure S4

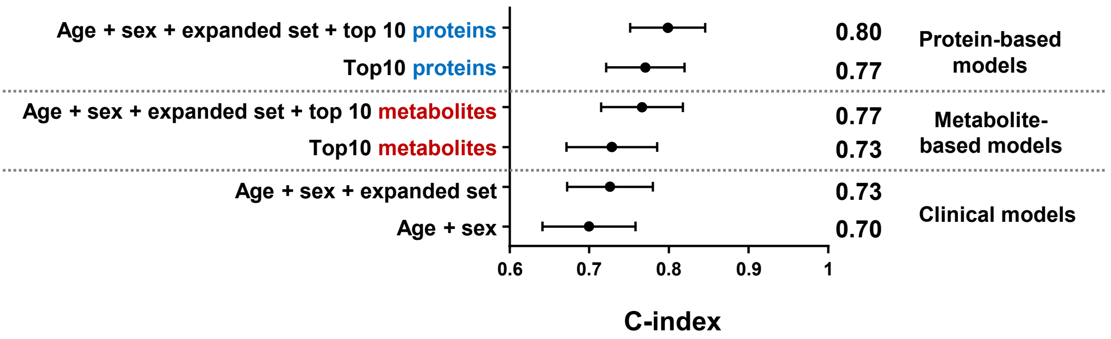

Figure S4. Performance comparison of Cox regression models incorporating the top 10 proteomic or metabolomic biomarkers.

Models were adjusted for age and sex, with additional models incorporating an expanded set of clinical covariates including educational attainment, alcohol consumption, smoking status, hypertension, and diabetes mellitus.
